# Interventional Protocol for Treatment of Complications after Esophagojejunostomy for Esophagogastric Carcinoma

**DOI:** 10.1155/2019/1465301

**Published:** 2019-12-01

**Authors:** Yonghua Bi, Jindong Li, Mengfei Yi, Zepeng Yu, Xinwei Han, Jianzhuang Ren

**Affiliations:** ^1^Department of Interventional Radiology, The First Affiliated Hospital of Zhengzhou University, Zhengzhou, China; ^2^Department of Thoracic Surgery, The First Affiliated Hospital of Zhengzhou University, Zhengzhou, China

## Abstract

**Background:**

Anastomotic stenosis and leakage are rare complications after esophagojejunostomy. The management of complications after esophagojejunostomy remains a challenge. We evaluated the outcomes and clinical effectiveness of an alternative interventional protocol.

**Objectives:**

To determine the safety and efficacy of interventional treatment for the management of complications after esophagojejunostomy.

**Methods:**

This study included 24 consecutive patients with complications after esophagojejunostomy treated using interventional protocol. Patients received balloon dilation or stenting for anastomotic stenosis. Patients with anastomotic leakage received three-tube placement or retrievable covered esophageal stent placement, followed by abscess drainage, nutritional support, and anti-inflammatory treatment. The three tubes and esophageal stents were removed after leakage healing and stenosis ceased.

**Results:**

Thirteen patients received three-tube method, and 16 patients received covered stent placement. All procedures were technically successful, except for a failure of Y-type esophageal stent placement in one patient. The median retention time of stent and abscess drainage tube was 67.5 days and 87 days, respectively. No perioperative death, esophageal rupture, or massive hemorrhage was found during procedures. During follow-up, 14 patients died of cancer recurrence, and one died of severe pulmonary infection. The 1-, 3-, 5-year survival rates were 39.5%, 23.7%, and 23.7%, respectively.

**Conclusion:**

Interventional protocol is safe, feasible, and efficacious for treatment of complications after esophagojejunostomy.

## 1. Introduction

Patients with resectable esophagogastric carcinoma are commonly treated with gastrectomy and esophagectomy [1, 2]. Complications including anastomotic stenosis and/or anastomotic leakage are rare after esophagojejunostomy [3], with an overall incidence of less than 3.0% [4]. Currently, various conservative treatments have been reported, including endoscopic transluminal drainage, stent placement, and biodegradable leakage plugs, or fibrin glue [5–8]. Endoscopic placement of covered stents has been used for the treatment of anastomotic leakages after esophagogastrostomy or esophagojejunostomy [9, 10]. However, treating complications after esophagojejunostomy remains challenging, and the optimal protocol has not been determined [2, 6, 11].

To date, only limited data are available on interventional treatment of anastomotic leakage or stenosis under fluoroscopic guidance. We used an interventional protocol consisting of balloon dilation for benign anastomotic stenosis, stent for malignant, and three-tube method with or without covered esophageal stent placement for anastomotic leakage. In this study, we aimed to determine the safety and efficacy of this protocol for treatment of complications after esophagojejunostomy.

## 2. Methods

### 2.1. Patient Selection

**Figure 1:**
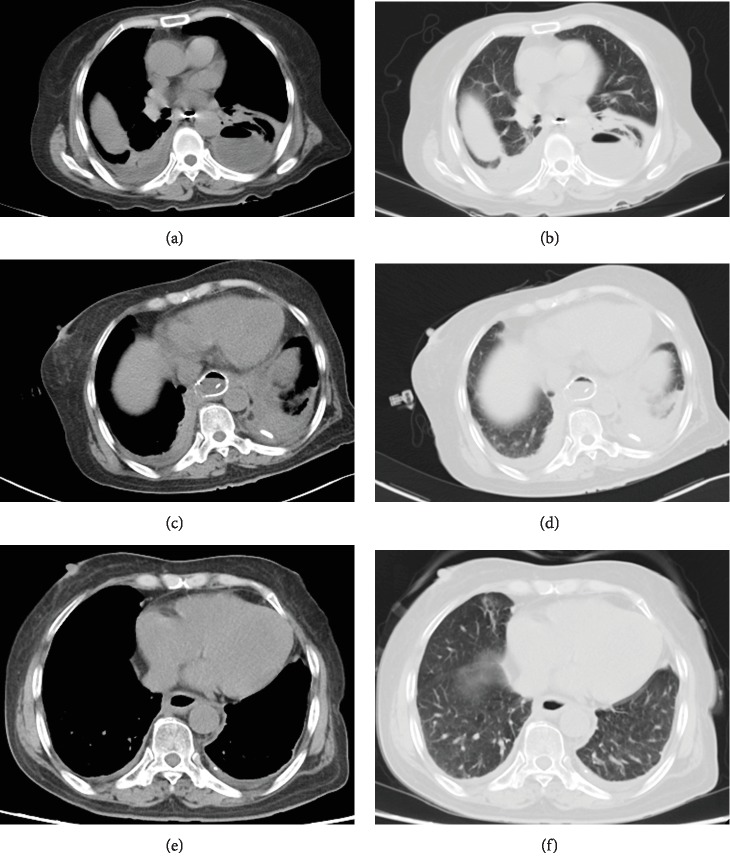
Chest computed tomography for case 16. (a, b) Chest CT scan in the mediastinal and lung windows shows anastomotic leakage, atelectasis, and bilateral pleural effusion before interventional procedure. (c, d) At one month after three-tube treatment and covered stent placement, a chest CT scan shows a covered stent with decreased pleural effusion. (e, f) After removal of three-tube and covered stent, a chest CT scan shows disappearance of abscess, expansion of the lungs with no pleural effusion.

This study was approved by the Ethics Committee Board of the First Affiliated Hospital of Zhengzhou University. All informed consents were obtained from the patients. This study enrolled all patients with anastomotic stenosis and/or anastomotic leakage who received interventional treatments in our department between May 2012 and February 2018. The diagnosis of complications after esophagojejunostomy was made based on finding of esophagography and the chest-computed tomography (Figures 1(a) and 1(b)). Patients with esophagotracheal fistula or spontaneous esophageal perforation were excluded. All patients were treated on an inpatient basis.

### 2.2. Three-Tube Method

**Figure 2:**
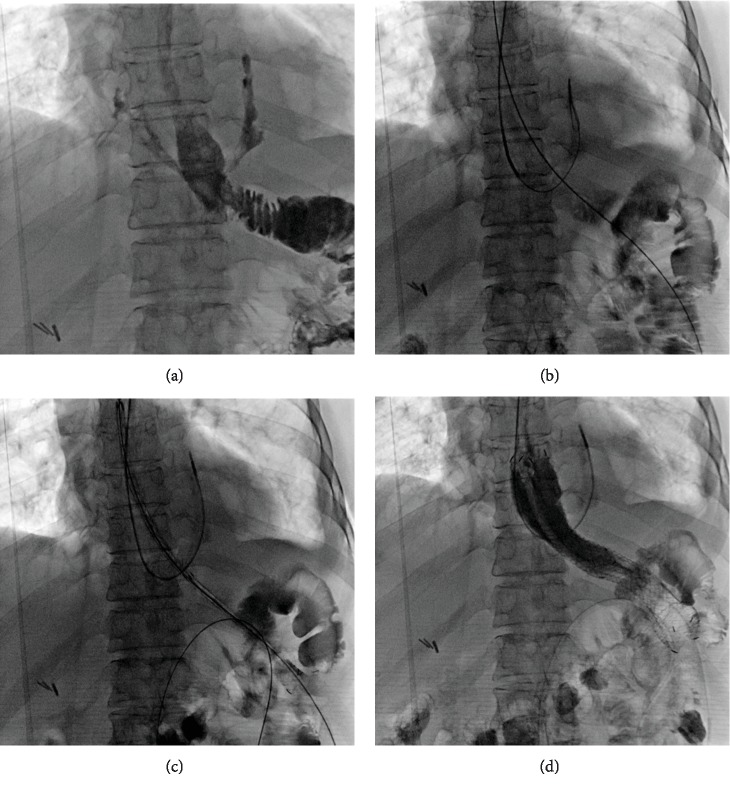
A 65-year-old woman with anastomotic leakage (case 16). (a) Esophagography showing the site of anastomotic leakage in the lower esophagus. (b) A 5 F catheter was introduced through the outlet of leakage into the distal end of the abscess cavity. (c) A stent delivery system was introduced along the stiff guidewire. (d) Esophagography showed that the contrast agent flowed through the stent with no leakage after esophageal stenting.

All interventional procedures were performed under fluoroscopic guidance and conscious sedation. After the esophagus was anesthetized by oral lidocaine gel, esophagography was performed to show the site of anastomotic leakage and stenosis (Figure 2(a)). A 5 F catheter was introduced through the outlet of anastomotic leakage into the distal end of the abscess cavity (Figure 2(b)). The catheter was then exchanged with a 5 F straight or pigtail catheter (Cook Medical, Inc., Bloomington, IN). Continuous negative pressure suction was used to achieve effective drainage of abscess cavity. A gastrointestinal decompression tube and jejunal feeding tube were inserted in the intestinal cavity of the anastomosis and the proximal jejunum, respectively.

### 2.3. Balloon Dilation and Esophageal Stent Placement

**Figure 3:**
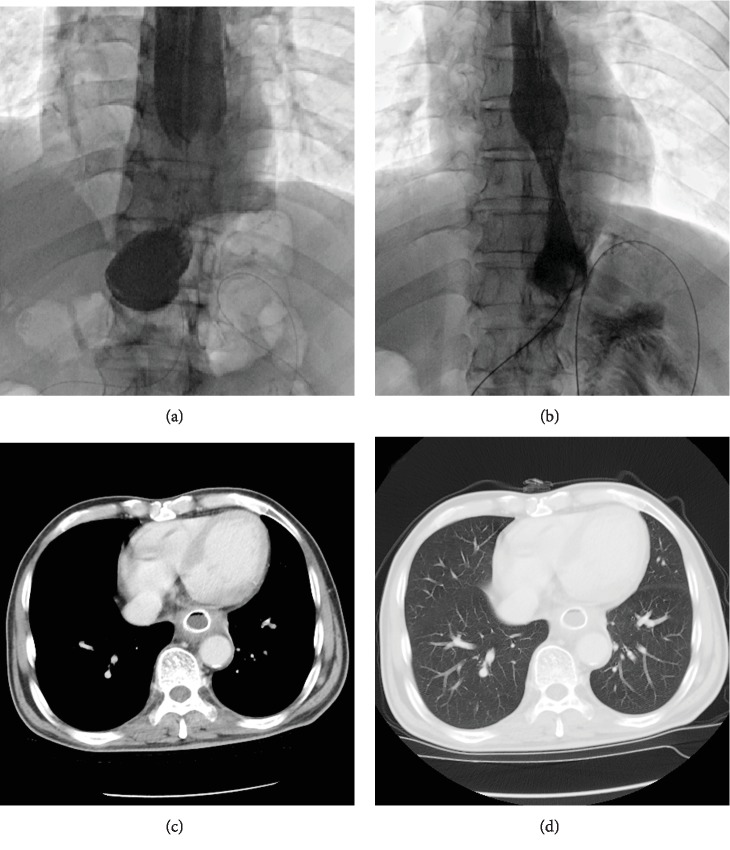
A 63-year-old man with anastomotic stenosis treated by stent placement (case 12). (a) Esophagography showed the site of anastomotic stenosis in the lower esophagus. (b) A covered stent was inserted. (c, d) At 48 days after stent placement, a chest CT scan shows well expansion of stent and relief of stenosis.

Balloon dilation was performed in patient with benign anastomotic stenosis (Figures 3(a) and 3(b)). The diameter of the stents ranges from 10 to 26 mm. The length of the stents ranges from 40 to 60 mm. The retrievable covered esophageal stent was used for the treatment of anastomotic leakage or malignant stenosis, and 2 Y type of intestinal stent were used (Nanjing Micro-Tech Medical Company, Nanjing, China). The stent diameter ranges from 18 to 22 mm. The stent length ranges from 80 to 140 mm. Adequate stent coverage is allowed on both sides of the stenosis or leakage to ensure complete coverage. A 5 F catheter was introduced into the gastral cavity, and a stiff guidewire was exchanged. A stent delivery system was introduced along the stiff guidewire and then adjusted and released slowly (Figure 2(c)). Esophagography was performed again to show change of anastomotic leak and stenosis (Figure 2(d)).

### 2.4. Postoperative Care

**Figure 4:**
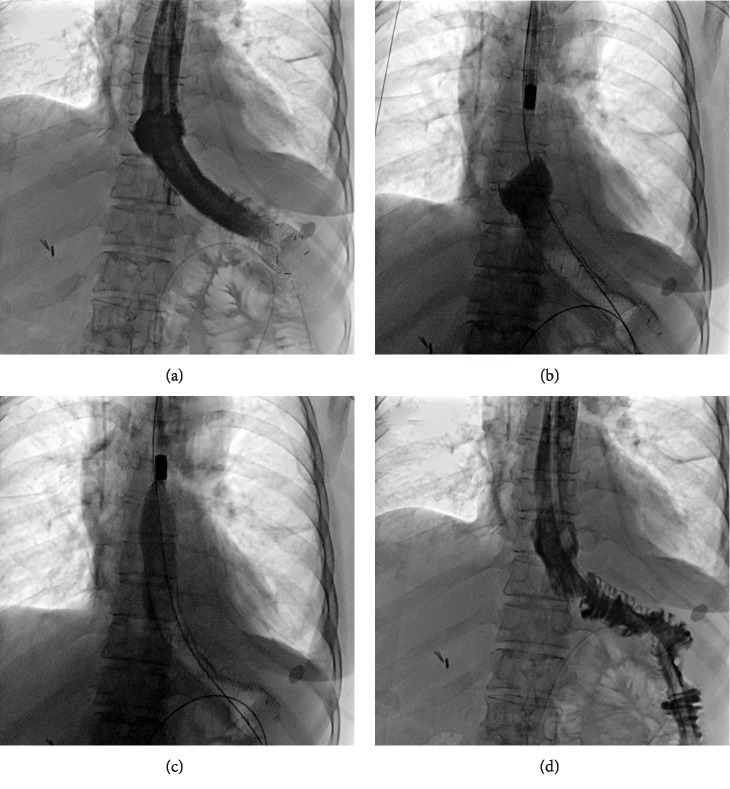
Stent removal for case 16. (a) Esophagography shows that the contrast agent flows though the covered stent with no leakage 45 days after stent placement. (b, c) The stent was removed under fluoroscopic guidance. (d) After removal of stent, esophagography showed that the contrast agent flowed through the esophagus with no leakage.

Enteral nutrition (Milupa Gmbh & Co. KG, Friedrichsdorf, Germany) was provided through the jejunal feeding tube. Patients were not permitted to oral feed until leakage sealing and stenosis relief was confirmed by esophagography. Broad-spectrum antibiotic treatment was used for patients with anastomotic leakage before and after procedure. The abscess cavity was irrigated twice a day by physiological saline via the drainage tube. Postoperative esophagography and chest CT were performed within 1 week to show the size change of abscess cavity and the position of drainage tube (Figures 1(c) and 1(d); Figures 3(c) and 3(d)). The covered stents and drainage tubes were removed if chest CT confirmed disappearance of abscess cavity and full expansion of the lungs (Figures 1(e) and 1(f); Figures 4(a)–4(d)).

## 3. Results

### 3.1. General Information

This study involved a total of 24 patients with complications after esophagojejunostomy, including 18 men and 6 women (Table 1). The ages of the patients ranged from 38 years to 74 years, with a median age of 64 years. The median disease course before referral to our department was 5.5 months (range: 0.3 to 72 months). The median interval between esophageal surgery and complications was 6.8 months (range: 0.2 to 52 months). There were 11 cases of anastomotic leakage, 11 cases of anastomotic stenosis, and 2 patients showed anastomotic leakage combined with stenosis.

### 3.2. Intervention Outcomes

Three-tube method was used for 13 patients with anastomotic leakage, of which, 7 patients received covered stent placement. For patients with anastomotic stenosis, 4 patients received balloon dilation, and 9 patients received stent placement. A total of 21 covered esophageal stents were placed for 16 patients, with a median diameter of 20 mm and median length of 100 mm. Except for failure of placement of Y-type intestinal stent in one patient due to complete occlusion, all operations were technically successful, with appropriate positions and satisfactory expansion of stents or drainage of tubes. For patients who received stent placement for anastomotic leakage, the leakage was completely blocked after stenting confirmed by immediate postprocedural esophagography. Besides, three patients received transcatheter arterial chemoembolization for cancer recurrence, one patient received lumbar sympathetic block for severe abdominal pain, and one patient received percutaneous transhepatic cholangial drainage for biliary obstruction. One colon stent was inserted, and one intestinal obstruction catheter was used for intestinal obstruction due to tumor migration.

### 3.3. Complications

**Figure 5:**
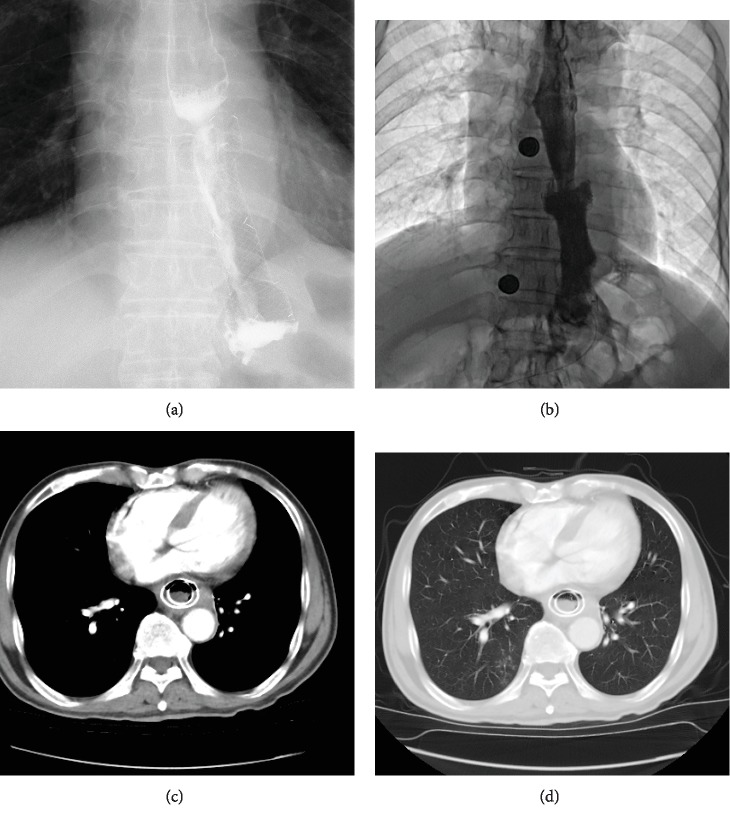
Additional stent placement for restenosis of stent (case 12). (a) Esophagography showed severe restenosis in the proximal end of stent 5 months later. (b) Another covered stent was inserted to relief restenosis. (c, d) A chest CT scan showed well expansion of stent and relief of restenosis.

No massive hemorrhage, esophageal rupture, or other complications occurred during procedures. Stent restenosis was found in 3 patients, and an additional stent was inserted for these patients (Figures 5(a)–5(d)). Two patients showed stent migration, and stents were adjusted. No migration of abscess drainage tube was found. The abscess drainage tubes were adjusted for 0 to 6 times.

### 3.4. Follow-Up

Removal of stent or abscess drainage tube was successfully performed for 8 patients. The median retention time of stent was 67.5 days (range, 17 to 390 s; Figures 4(c) and 4(d)). The median retention time of abscess drainage tube was 87 days (range, 7 to 241 days). Except one patient lost to follow-up, 23 patients were followed up for a median time of 6.8 months (range: 0.2 to 52.0 months). At this time, 8 patients were still alive, who were able to return to their normal living conditions without any symptom. During follow-up, 14 patients died of cancer recurrence, and one died of pulmonary infection. The 1-, 3-, and 5-year survival rates were 39.5%, 23.7%, and 23.7%, respectively.

## 4. Discussion

Anastomotic stenosis and anastomotic leakage are rare complications after esophagojejunostomy [3]. The overall incidence of anastomotic leakage and stenosis after esophagojejunostomy was 2.1%-3.0% and 2.7%-2.9%, respectively [4]. Management of complications after esophagojejunostomy is still challenging with no optimal treatment protocol [2, 6, 11]. Surgical repair was the traditional protocol [12], and various conservative treatment protocols have been reported, including endoscopic transluminal drainage/clipping, stent placement, biodegradable leakage plugs, or fibrin glue [5–8]. Esophageal stent was initially used as a palliative treatment for malignant dysphagia. Recently, esophageal stents have expanded to treat benign disease [13, 14], including plastic stents [15–17] and metal stents [13, 18, 19]. Esophageal metallic stent placement may serve as an alternative protocol for complications after esophagojejunostomy. Metallic stent has been used for treatment of malignant stenosis or perforation [18, 19], which is also used for the treatment of benign diseases in recent years, such as anastomotic leakage or stenosis [13]. In this study, a total of 21 covered esophageal stents were placed in 7 patients with anastomotic leakage, with a median retention time of 67.5 days.

Successful management of anastomotic leakage after esophagojejunostomy requires adequate therapy of the associated infection and effective elimination of contamination by prompt placement of the covered stent. We present 24 consecutive patients treated with interventional protocol for complications after esophagojejunostomy. We found that interventional protocol can be easily and effectively performed under fluoroscopic guidance with a high technical success rate and no severe complications. No perioperative death was observed in this study, which was lower than previous reports [15, 16, 20, 21]. The gastral cavity is still allowed to drain by a drainage tube after placement of a covered stent. The median retention time of abscess drainage tube was 87 days.

There were certain complications of the interventional protocol. Stent migration is a common complication, especially in patients without luminal stenosis [22, 23]. Covered stents were used in this study; stent migration was found in 2 patients and was adjusted effectively. Stent restenosis was found in 3 patients, and an additional stent was inserted for these patients. The drainage tube was exchanged or adjusted for 0 to 6 times.

There are some limitations in this study. This is a retrospective study with a relatively small number of patients. The healing time required for anastomotic leakage is long; however, the esophageal stents are usually removed within 3 months to avoid long-term complications.

## 5. Conclusions

Our study shows that interventional treatment of complications after esophagojejunostomy can be considered a safe and effective alternative protocol. Combination of interventional treatment and additional supportive therapy is essential for anastomotic leakage.

## Figures and Tables

**(a):** 

No.	Gender	Age(Y)	Cause	Course of disease (M)	Duration from surgery to stenosis/leak (M)	Duration of hospitalization (D)	Type of disease	Comorbidities	Interventional methods	Stent size (mm × mm)
1	Male	70	Gastric carcinoma	9.0	8.5	16	Anastomotic stenosis	None	Stent	18 × 120
2	Male	64	Gastric carcinoma	7.0	3.0	6	Anastomotic stenosis	None	Balloon+stent	20 × 120
3	Male	72	Gastric carcinoma	5.0	2.0	35	Anastomotic stenosis	Hypertension	Balloon+stent	22 × 120; 20 × 100; 20 × 100; 20 × 100
4	Female	66	Gastric carcinoma	1.0	1.0	32	Anastomotic stenosis	Hypertension+cardiac insufficiency	Balloon	None
5	Male	74	Gastric carcinoma	17.0	13.0	13	Anastomotic stenosis	None	Stent	20 × 120
6	Male	56	Esophagogastric carcinoma	12.0	10.0	7	Anastomotic stenosis	None	Stent	20 × 100
7	Male	67	Carcinoma of gastric cardia	3.0	7.0	28	Anastomotic stenosis	Hypertension	Stent	18 × 120
8	Female	69	Gastric carcinoma	0.7	0.7	40	Anastomotic stenosis	None	Balloon	None
9	Male	65	Carcinoma of gastric cardia	24.0	20.0	17	Anastomotic stenosis	None	Stent	20 × 120
10	Male	55	Gastric carcinoma	12.0	11.0	22	Anastomotic stenosis	Diabetes	Stent	20 × 100; 20 × 120
11	Male	58	Gastric carcinoma	7.0	6.5	22	Anastomotic stenosis	None	Stent	18 × 100
12	Male	63	Gastric carcinoma	4.0	1.2	16	Anastomotic stenosis+leak	None	Stent+three-tube method	20 × 100; 20 × 100
13	Female	51	Gastric carcinoma	11.0	9.0	36	Anastomotic stenosis+leak	None	Stent+three-tube method	22 × 80 − 22 × 50 − 22 × 50
14	Male	44	Gastric carcinoma	4.0	1.0	21	Anastomotic leak	None	Three-tube method	None
15	Female	72	Gastric carcinoma	0.3	0.2	28	Anastomotic leak	None	Stent+three-tube method	20 × 100
16	Female	65	Gastric carcinoma	6.0	0.6	75	Anastomotic leak	Diabetes	Stent+three-tube method	20 × 140
17	Female	38	Gastric carcinoma	1.0	0.3	48	Anastomotic leak	None	Three-tube method	None
18	Male	60	Gastric carcinoma	4.0	0.1	18	Anastomotic leak	None	Three-tube method	None
19	Male	43	Carcinoma of gastric cardia	3.0	2.3	41	Anastomotic leak	None	Stent+three-tube method	20 × 120
20	Male	68	Gastric carcinoma	0.7	0.1	13	Anastomotic leak	None	Three-tube method	None
21	Male	64	Carcinoma of gastric cardia	3.0	0.2	32	Anastomotic leak	None	Stent+three-tube method	20 × 100 − 20 × 40 − 20 × 40
22	Male	53	Gastric carcinoma	6.0	1.8	19	Anastomotic leak	None	Three-tube method	None
23	Male	69	Gastric carcinoma	72.0	0.4	53	Anastomotic leak	Hypertension	Three-tube method	None
24	Male	53	Gastric carcinoma	6.0	0.3	32	Anastomotic leak	None	Stent+three-tube method	20 × 100

**(b):** 

Balloon size (mm × mm)	Complications	Adjustment time of stent	Adjustment time of abscess drainage tube	Removal of stent/abscess drainage tube	Retention days of stent	Retention days of abscess drainage tube	Other interventional treatments	Survival time after stent implantation (M)	Death cause
None	None	0	0	Yes	55	—	None	6.0	Died of cancer recurrence
16 × 40	None	0	0	No	390	—	None	13.0	Died of cancer recurrence
16 × 40; 18 × 40	Stent restenosis	0	0	No	368	—	TACE	12.2	Died of cancer recurrence
16 × 40; 18 × 40; 26 × 40	None	0	0	—	—	—	None	10.2	Survive without symptom
None	None	0	0	No	344	—	None	11.4	Survive without symptom
None	None	0	0	Lost to follow-up	Lost to follow-up	—	None	—	Lost to follow-up
None	None	0	0	No	73	—	TACE	2.4	Died of cancer recurrence
18 × 60; 18 × 60; 18 × 60	None	0	0	—	—	—	None	10.3	Survive without symptom
None	None	0	0	No	120	—	Lumbar sympathetic block	4.0	Died of cancer recurrence
None	Stent restenosis	0	0	No	71	—	TACE+intestinal obstruction catheter	2.3	Died of cancer recurrence
None	None	0	0	No	50	—	None	1.6	Died of cancer recurrence
None	Stent restenosis	0	0	No	30	30	None	6.8	Survive without symptom
10 × 40; 10 × 40	None	0	2	No	—	41	None	1.3	Died of cancer recurrence
None	None	0	0	Yes	—	103	None	46.9	Survive without symptom
None	None	0	2	Yes	17	17	None	2.1	Died of cancer recurrence
None	Stent migration	1	0	Yes	45	45	PTCD+stenting in colon	19.9	Died of cancer recurrence
None	None	0	0	No	—	177	None	5.9	Died of cancer recurrence
None	None	0	0	Yes	—	241	None	52.0	Survive without symptom after surgery repair
None	Stent migration	1	2	No	38	38	None	1.2	Died of pulmonary infection
None	None	0	0	No	—	7	None	0.2	Died of cancer recurrence
None	None	0	2	No	87	87	None	2.9	Died of cancer recurrence
None	None	0	0	Yes	—	212	None	48.0	Survive without symptom
None	None	0	6	Yes	—	111	None	10.6	Survive without symptom
None	None	0	4	Yes	64	178	None	8.1	Died of cancer recurrence

Y: years; M: months; D: days; TACE: transcatheter arterial chemoembolization; PTCD: percutaneous transhepatic cholangial drainage.

## Data Availability

The data used to support the findings of this study are available from the corresponding author upon request.
